# SEMPro: A Data-Driven Pipeline To Learn Structure–Property Insights from Scanning Electron Microscopy Images

**DOI:** 10.1021/acsmaterialslett.3c00909

**Published:** 2023-10-24

**Authors:** Brandon Ho, Jiayu Zhao, Joseph Liu, Lisa Tang, Zhecun Guan, Xiao Li, Minghao Li, Elizabeth Howard, Rebecca Wheeler, Jinhye Bae

**Affiliations:** † Department of NanoEngineering, University of California San Diego, La Jolla, California 92093, United States; ‡ Chemical Engineering Program, University of California San Diego, La Jolla, California 92093, United States; § Material Science and Engineering Program, University of California San Diego, La Jolla, California 92093, United States; ∥ Department of Electrical and Computer Engineering, University of California San Diego, La Jolla, California 92093, United States

## Abstract

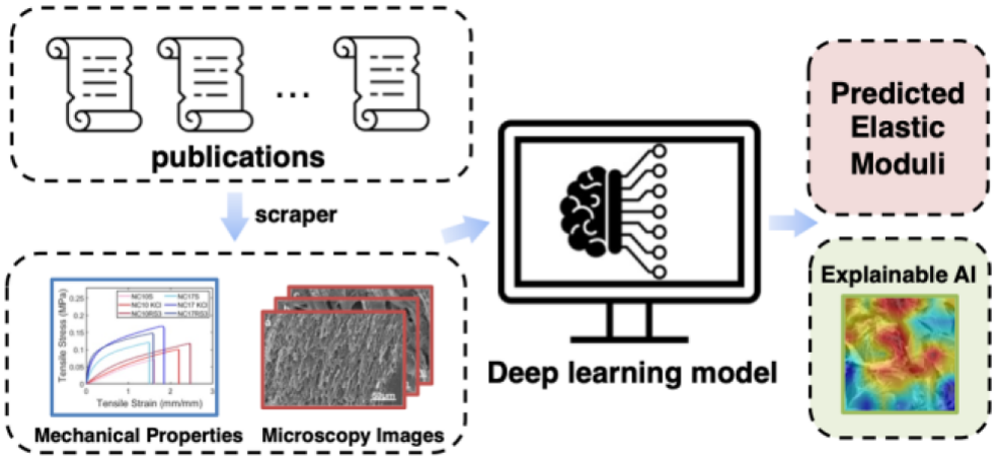

Analyzing hydrogel microstructure through scanning electron microscopy (SEM) images is crucial in understanding hydrogel properties. However, the analysis of SEM images in hydrogel research heavily relies on the intuition of individual researchers and is constrained by the limited size of the dataset. To address this, we propose SEMPro, a data-driven solution using web-scraping and deep learning (DL) to compile and analyze the structure–property relationships of hydrogels through SEM images. It accurately predicts the elastic modulus from SEM images within the same order of magnitude and displays a learned extraction of modulus-relevant features in SEM images as seen through the nontrivial activation mapping and transfer learning. By employing Explainable AI through activation map exposure, SEMPro validates the model predictions. SEMPro represents a closed-loop data collection and analysis pipeline, providing critical insights into hydrogels and soft materials. This innovative approach has the potential to revolutionize hydrogel research, offering high-dimensional insights for further advancements.

Understanding mechanical properties is crucial in materials research and development as it allows researchers and engineers to select the most suitable materials for realizing specific properties and applications. Hydrogels, composed of a complex, three-dimensional cross-linked hydrophilic polymer network that can absorb and retain a large amount of water (70%–99%),^1^ are one of the most commonly used materials for applications in biomedical engineering,^2^ drug delivery systems,^3^ tissue engineering,^4,5^ and wound dressings^6^ because of their distinctive characteristics such as high biocompatibility, soft and elastic nature, and similarity to natural tissues.^7^ However, conventional hydrogels are considered fragile materials that severely limit their scope of applications. In recent decades, substantial efforts have been dedicated to improving the mechanical properties of hydrogels, paving the way for the development of ionic skins,^8^ wearable sensors,^9^ and soft robots^10^ that can effortlessly adapt to changing demands, aligning with the advancements of the fourth industrial revolution. Several fabrication techniques have been employed to create hydrogels with tunable mechanical properties by changing their inner porous structures, such as the freeze–thaw method,^11^ solvent casting and particulate leaching,^12^ gas foaming,^13^ and phase separation.^14^ These methods enable control over the internal pore size or porosity by adjusting experiment parameters (i.e., freezing time, sacrificial particle size, gas pressure, and incubation time in a phase-separated state), thus allowing for the tuning of mechanical properties. Furthermore, the mechanical properties can be significantly improved by employing strategies that involve creating interpenetrating polymer networks,^15,16^ making long polymer chains with a significantly high degree of entanglement compared to cross-links,^17^ incorporating nanoparticles to form percolating networks,^18−21^ or harnessing the electrostatic interactions in polyampholytes.^22^ As a result, these techniques have a direct impact on changes in the microstructure of hydrogel networks, providing valuable insight into their mechanical properties.

It is worth noting that while the mechanical properties of a hydrogel are affected by its internal structure in three dimensions, researchers have relied mostly on data extracted from two-dimensional (2D) images such as pore size,^23^ thickness,^24^ and density,^20^ to support the interpretations of mechanical properties of hydrogels. This indicates that there is a reasonable basis for interpreting the relationship between the 2D porous structure of a hydrogel and its mechanical properties. To examine the porous structure of hydrogels, scanning electron microscopy (SEM) imaging has emerged as an important tool with high spatial resolution, leading to a better understanding of the mechanical properties of hydrogels. However, as mentioned above, the quantitative analysis of the information present in SEM image data has primarily been limited to 2D metrics. To fully unlock the potential of SEM images, we propose employing deep learning techniques to investigate the relationship between microstructure and mechanical properties. Deep learning, notably through the application of multilayer perceptron models, has proven to be an effective statistical method for the automated analysis of high-dimensional data in the realm of soft materials.^25,26^ Combining dense connections between learned parameters and nonlinear activation functions allows deep neural networks (DNNs) to accurately model complex relationships across various data domains.^27^

With respect to image processing, convolutional neural networks (CNNs) have demonstrated their effectiveness in learning from images due to their inherent ability to recognize local patterns and translation invariance.^28^ Prior research has shown CNNs to be effective in predicting stress–strain curves from second harmonic generation (SHG) images of collagenous tissues.^29^ Olenskyj et al. reported the prediction of compression curves from micro-computational tomography (micro-CT) images using deep learning.^30^ While these approaches produced a relatively low prediction error (10.9%–13.2%), it remains unknown if these models can be generalized to other materials since these studies only tested on one type of material, using a small dataset (less than 100 samples).

It is widely acknowledged that the accuracy of a deep learning model is significantly influenced by the size and quality of its training dataset.^31^ However, this poses a unique challenge due to the substantial cost, both monetary and temporal, associated with collecting SEM images for the individual project. While there are publicly available SEM datasets such as the UltraHigh Carbon Steel Micrograph DataBase^32^ and the Aversa SEM dataset,^33^ to the best of our knowledge, there is currently no existing dataset that encompasses SEM images along with their corresponding properties for soft materials, specifically hydrogels. This absence of a comprehensive dataset presents a significant obstacle in the exploration of advanced materials within the soft materials community.

In this work, we develop and optimize a low-cost, semiautomatic data collection and analysis pipeline to learn structure–property insights from SEM images (SEMPro; see Figure 1). To the best of our knowledge, this is the first report of using deep learning models to predict elastic moduli from SEM images of hydrogels. Modern data-driven research methods point to automated web-scraping and natural language processing (NLP) as a method to supplement smaller datasets in microscopy and materials synthesis.^34−36^ Web scraping is the process of automatically extracting website metadata from its HTML source code. Combined with natural language processing (NLP), a method of automating natural language analysis to understand and subsequently extract relevant metrics, we compile a large amount of SEM images from the online, public repository of research papers with permission (Figure 1a). After cleaning and labeling data, we experiment with the state-of-art CNN architecture and learning parameters to optimize learning for our task set and dataset (Figure 1b). The accuracy of the generalized model can be tailored to a target hydrogel with transfer learning,^37^ which is a method of training a network primarily on a larger, more generalized dataset and then secondarily on the target predictive task, which allows for the application of deep learning on smaller datasets of interest.^38,39^ We also investigate how regression activation mapping can give feedback on the quality of learning and fuel model optimization. By using Explainable AI methods, in contrast to the classical deep learning black box, we show that it is possible to optimize CNNs toward learning the mechanical insights of hydrogels to aid researchers in microstructure analysis (Figure 1c).

**Figure 1:**
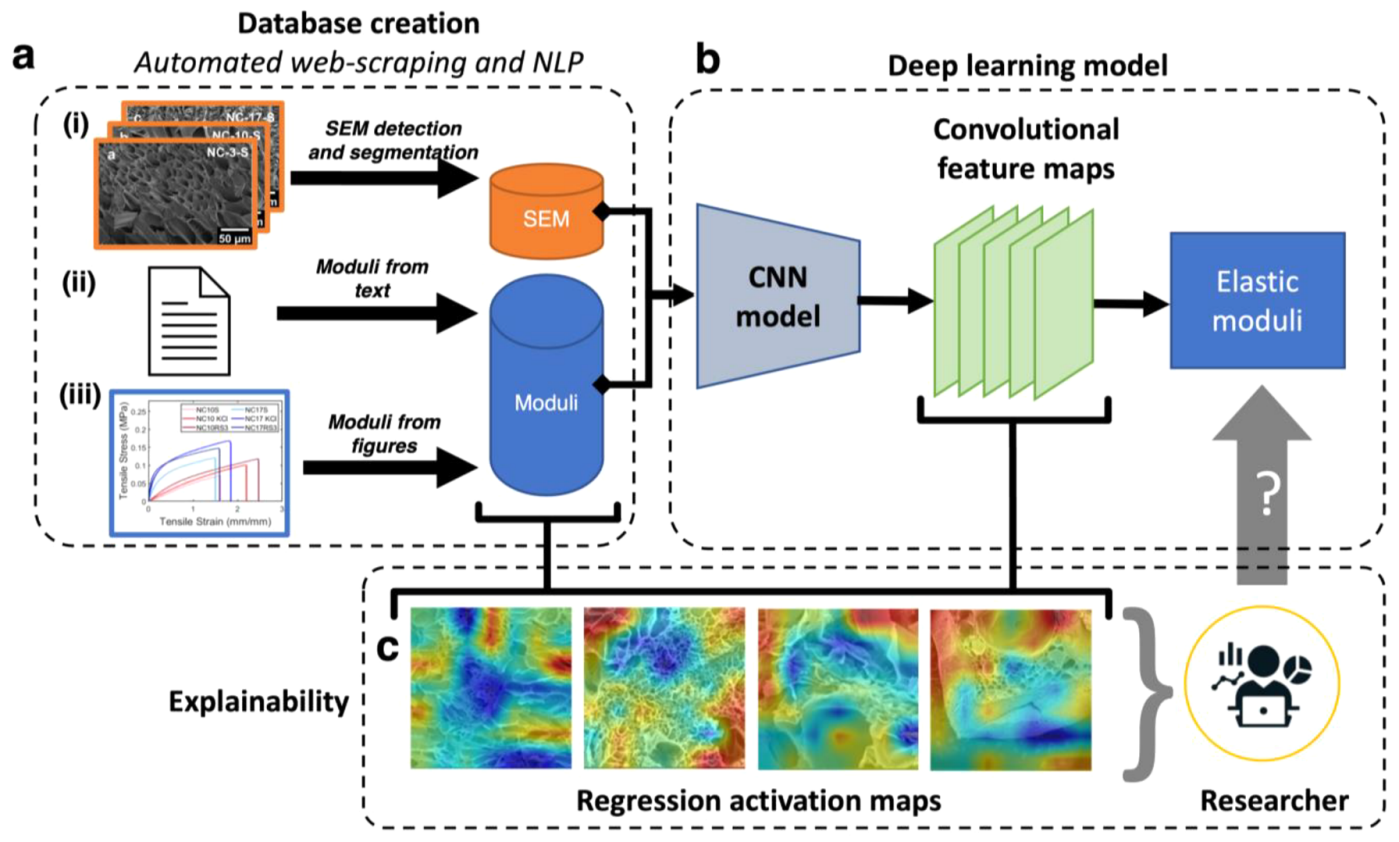
(a) Schematic representation of SEMPro dataset creation workflow: (i) SEM images are segmented from the figures of the research paper and modulus is extracted from (ii) paper text and (iii) stress/strain curves, which are then stored in two individual databases. The representative SEM images and stress/strain curves shown in (i) and (iii), respectively, are from our previous work.^21^ (b) Entries from both databases are then matched and validated by researchers to form the labeled SEM training dataset that feeds into the deep learning workflow. The CNN model takes in an input SEM image, computes feature maps from convolving learned filters over the image, and predicts the elastic modulus from the feature maps. (c) The prediction of the elastic modulus on a given input SEM is explained through feature maps overlaid on the input image. These regression activation maps nontrivially highlight structural regions on the hydrogel with higher (red) and lower (blue) weights of relevancy to elastic modulus prediction. The researcher can then query the activation maps to understand the predicted modulus from the model, as well as validate model learning and generalizability.

We develop a method of obtaining SEM images and elastic moduli datasets from research papers through our web scraping and data filtering pipeline, SEMPro-Scraper, written in Python. The scraping functionality of SEMPro-Scraper is built off a boilerplate template from PaperScraper of VCU NLP Lab.^40^ Augmenting the web scraping utility of PaperScraper, SEMPro-Scraper includes general improvements in functionality in the form of support for extracting figure sets and data from supporting documentation. SEMPro-Scraper also performs analytical filtering of the website source code to search for SEM images and modulus-related data using research paper metadata. All researched papers scraped are sourced from the American Chemical Society (ACS), with permission to compile data from the former.

We first use BeautifulSoup4 (BS4)^41^ and Selenium Python packages to compile a list of research paper links from the keyword “hydrogel” through the query engine of ACS. Using BS4, we then extract the metadata of each web journal from its HTML source code. We subsequently pass the metadata through our data extraction pipeline.

The SEM image data are identified and extracted through NLP and automated image classification using the state-of-the-art object detection model, You Only Look Once (YOLO)^42^ (Figure 1a(i)). We used the fifth version of YOLO, YOLOv5. First, the figure caption is tokenized by subfigure letters and preliminarily evaluated for relevance by performing an iterative search for SEM-related keywords. This list of keywords is precompiled through a heuristic survey of unique words found in the description of SEM figures. If the figure caption contains keywords relevant to SEM, the figure is then passed to our SEM image detection model to be segmented into individual SEM images. The model was trained on a set of 150 manually labeled SEM figures to recognize and compute bounding boxes around each SEM image within a figure. Given the bounding boxes, we used the OpenCV^43^ image processing library to crop out specific SEM images from the figure. Individual images are saved in a directory with all other SEMs from the research papers. The corresponding figures are also saved for ease of validation of the scraped SEMs.

Hydrogel descriptors and modulus data are scraped both from the body (Figure 1a(ii)) and figures (Figure 1a(iii)) of each research paper through NLP. Sentences in the body and figure captions of the research paper are tokenized and passed through a relevant keyword search which evaluates whether the sentence pertains to modulus. This relevant keyword search is also based on a precompiled list of elastic modulus and keywords surrounding modulus values (e.g., Pa). If the body sentence contains one of the keywords, a sequential heuristic NLP model is implemented to extract hydrogel descriptors, such as the name and polymer composition for the referred hydrogel, as well as modulus values. Similarly, if a figure caption is considered relevant to the modulus, the figure is stored for modulus value extraction.

A single scraping session can be customized by keyword topics of the research papers scraped as well as the number of research papers to scrape. Each scrape ranges from 5 s to 1 min, depending on content relevance, with an average runtime of 20 s per research paper scraped. A cache is also logged during the scraping process to avoid the runtime cost of rescraping a research paper in the event of errors, cancellations, or future iterations. The final corpus of data was scraped from over 2000 research papers within ∼11.1 h.

The results of a scraping session are automatically compiled and logged into two CSV files: the modulus and the SEM image spreadsheet. As a prevalidation check, only research papers that contain both modulus and SEM image data are written to the final spreadsheets. Each row of the compiled SEM image spreadsheet represents a single SEM image and contains the link, title, figure number, letter, hydrogel, caption, figure path, and the respective segmented SEM image directory. The figure path and SEM image directory are also hyperlinked with an Excel macro for ease of access during SEM image selection and validation. The modulus file contains a collection of all modulus data collected for each valid research paper. Each row contains the research paper name, the hydrogel name, the contextual sentence from which the modulus was extracted, and the modulus in value or figure. For modulus values obtained from the body, the modulus is simply written. For modulus values embedded in figures, a hyperlink is provided to the referred figure in the dataset directory.

Using the two final spreadsheets of SEM and modulus data, a team of researchers selected, matched, and validated the SEM and modulus data to create the SEMPro dataset. The SEMPro dataset consists of 830 labeled SEM images of 553 unique hydrogels including both pure and composite hydrogels. The primary parameters used for training were the SEM image and the modulus value.

We used the state-of-the-art image classification model ResNeXt50^44^ as our CNN model with a small modification, where we replaced the output classification layer with a linear layer to output a single numerical value as the predicted elastic modulus. The model, referred to as the baseplate model in our following discussion, is programmed in Python with the PyTorch package and trained on the SEMPro dataset. Its performance was evaluated based on the coefficient of determination (*R*^2^) and mean absolute error (MAE). The data are loaded into a PyTorch dataset object with each sample represented as a tuple of SEM image, log modulus, and learning weight. For each image, the loss function is then weighted with the inverse of the smoothed distribution at the elastic modulus label.

Next, the dataset is split into a 5:1 train-test split, with a 4:1 train-validation split within the training set. The validation set performance is passed to a plateau learning rate scheduler to decay learning rate as the model converges. Our baseplate model was trained for 100 epochs with a batch size of 10 and learning rate of 10^–6^. The runtime of each epoch of training and validation was, on average, 20 s, and the model converged within ∼30 epochs. All models were trained on a GeForce GTX 1650.

To clean the images of extraneous data, such as peripheral text or graphics, and normalize the dataset, each raw SEM image was resized and cropped at the center to remove the scale bar with a final size of 224 × 224 pixels. For the training set, the images were randomly rotated and flipped for rotational invariance during learning. Label distribution smoothing (LDS) was performed by convolving a symmetric kernel with the empirical density distribution, LDS extracts a kernel-smoothed version that considers the information overlap among data samples of neighboring labels.^45^ The effective label density distribution is computed by LDS:
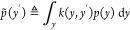
where *p*(*y*) is the number of appearances of the label of *y* in the training data, and  is the effective density of the label ,  is a symmetric kernel that satisfies  =  and . We used the Gaussian kernel as our symmetric kernel for the computation.

Paired *t*-test was conducted as a statistical method to compare the mean of the absolute errors generated by our baseplate model and two trivial models (i.e., mean and normal), in which the mean model always predicts the mean value of elastic moduli of the training set, and the normal model randomly predicts value from a normal distribution constructed based on the mean and standard deviation of the moduli in the training set. The confidence threshold for the paired *t*-testing was set to 0.05.

The following evaluation metrics have been applied to evaluate the performance of the model:(1) Coefficient of Determination (*R*^2^):
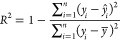
where *y̅* is defined as
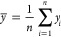
and *y*_*i*_ is the actual elastic modulus value,  is the predicted elastic modulus value, and *n* is the number of samples.

(2) Mean Absolute Error (MAE):
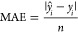
We also used *k*-folds cross-validation to evaluate our model performance. We first partition the dataset into *k* distinct sections. We then train *k* models, whereby, for each model, we uniquely assign one of the *k* partitions as a testing set while the remainder of the dataset is used for training.

Viability for transfer learning was evaluated at a wholistic and partial level on a smaller dataset (343 images) based on the SEM images of a nanocomposite hydrogel composed of different concentrations of graphene oxide and poly(*N*-isopropylacrylamide).^46^ We will name this dataset GO-PNIPAM in the discussion for transfer learning. In both experiments, LDS was disabled due to the smaller dataset size and even distribution of moduli. The learning rate was increased to 5 × 10^–4^ for faster convergence. All other training parameters remained the same as in the baseplate model. In the wholistic experiment, the full baseplate model was trained on the GO-PNIPAM dataset, with an average runtime of 7 s per epoch. In the partial transfer learning experiment, the convolutional layers of the ResNeXt50 model were frozen by disabling PyTorch autograd and only the linear output layer was trained. The average runtime of the partial transfer learning experiment was 5 s per epoch. Both converged within ∼10 epochs.

The saliency maps were made using an open-source PyTorch library for CAM methods.^47^ We chose to use the output feature maps from the final convolutional layer as the activation map overlay.

The elastic moduli in our SEMPro dataset are imbalanced, with most data being centered at ∼10^4^ Pa (Figure 2a). This agrees with the mechanical properties generally possessed by hydrogels.^48^ However, the imbalanced dataset implicitly biases the model toward the majority moduli label, which may lead to poor generalization on novel data, especially for the minority moduli labels. To address the issue of data underrepresentation, we used an LDS method developed by Yang et al.^45^ LDS promotes the utilization of kernel density estimation to capture the effective imbalance in datasets associated with continuous targets. Figure 2b shows the elastic moduli distribution after LDS, which exhibits the effective distribution by convolving a Gaussian kernel with the empirical density. We then use the effective label density to inversely weight the loss function based on moduli label representation, which subsequently increases learning on minority labels during training. The model trained with the dataset using LDS (Figure 2c) demonstrates higher precision, compared to the model trained without LDS (Figure 2d), as seen by the predictions of elastic moduli in the former model exhibiting a lower absolute error. Thus, unless specifically stated otherwise, we apply LDS to the dataset for all subsequent experiments.

**Figure 2:**
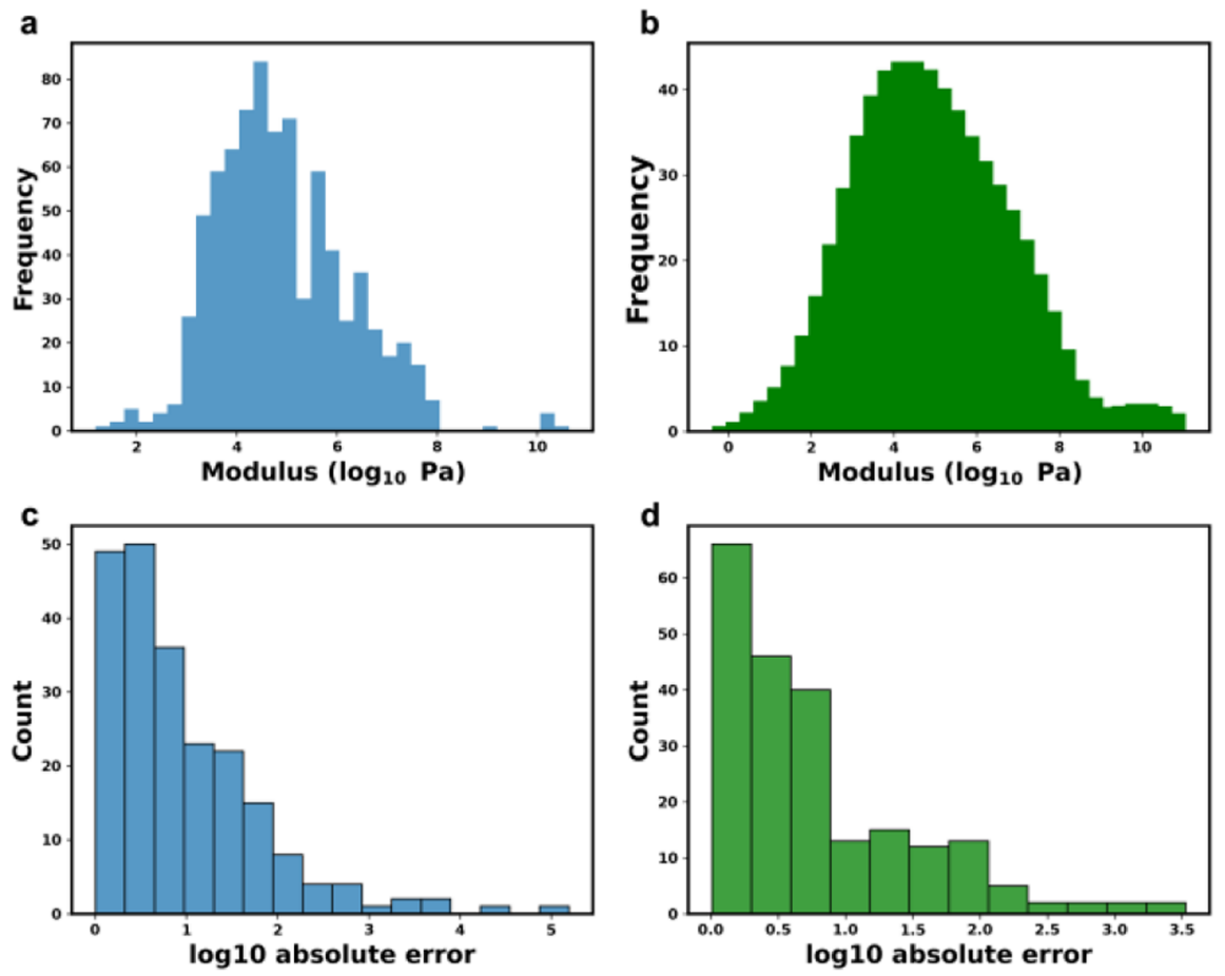
Distributions of modulus in SEMPro dataset (a) before and (b) after LDS smoothing. The corresponding distribution of log_10_ absolute error of training using the SEMPro dataset (c) before and (d) after LDS smoothing, respectively.

The predicted elastic moduli from the baseplate model on the SEMPro dataset are plotted against the actual elastic moduli for the train, test, and validation set, represented by the orange, blue, and green scatters, respectively (see Figure 3). The MAE of the baseplate model is 0.789, 0.792, and 0.807 for train, validation, and test, respectively. The corresponding coefficient of determination R^2^ is 0.597, 0.489, and 0.378 for the train, validation, and test, respectively (see Table 1). As there are no known pre-existing deep learning models predicting elastic moduli from SEM images of hydrogels, we also evaluate our model against two trivial models: one that predicts the mean of the training dataset for every image and one that randomly chooses from a normal distribution modeled after the training dataset. Each model is trained on a randomly generated training set and evaluated against a test set that it has not seen during training. The distributions of the evaluation results, given by the absolute error between the predicted and expected log moduli, are shown in Figure 4. The baseplate model exhibits the lowest median value of absolute error (0.55 log Pa), while the ones produced by the mean and normal models are 0.85 and 1.25 log Pa, respectively. We compute paired *t*-tests between the baseplate model and the mean and normal models with an alternative hypothesis stating that the baseplate model error distribution has a mean that is less than the trivial models. The results show confidently that we can reject the null hypothesis and, consequently, that the baseplate model performs better than these two trivial models. Our model thus displays a learned, nontrivial relationship between the SEM images and the elastic moduli of hydrogels.

**Figure 3:**
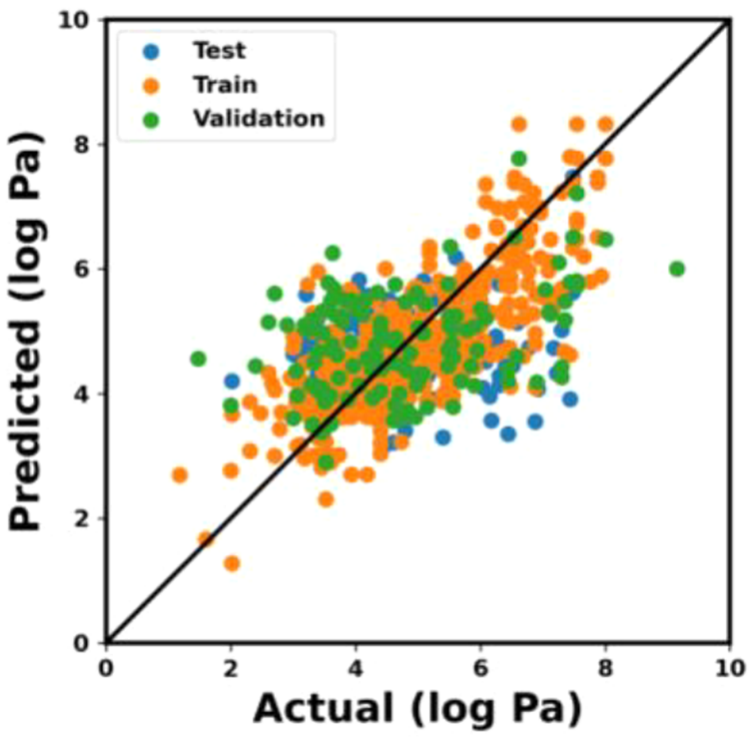
Elastic moduli predicted using baseplate model plotted against the actual elastic moduli for the train (orange), test (blue), and validation (green) sets, respectively.

**Table 1:** Evaluation Metrics Including Mean Absolute Error (MAE) and Coefficient of Determination (*R*^2^) for Predicting Elastic Modulus of Machine Learning Models

	MAE			*R*^2^		
ML model	train	validation	test	train	validation	test
baseplate	0.789	0.792	0.807	0.597	0.489	0.378

**Figure 4:**
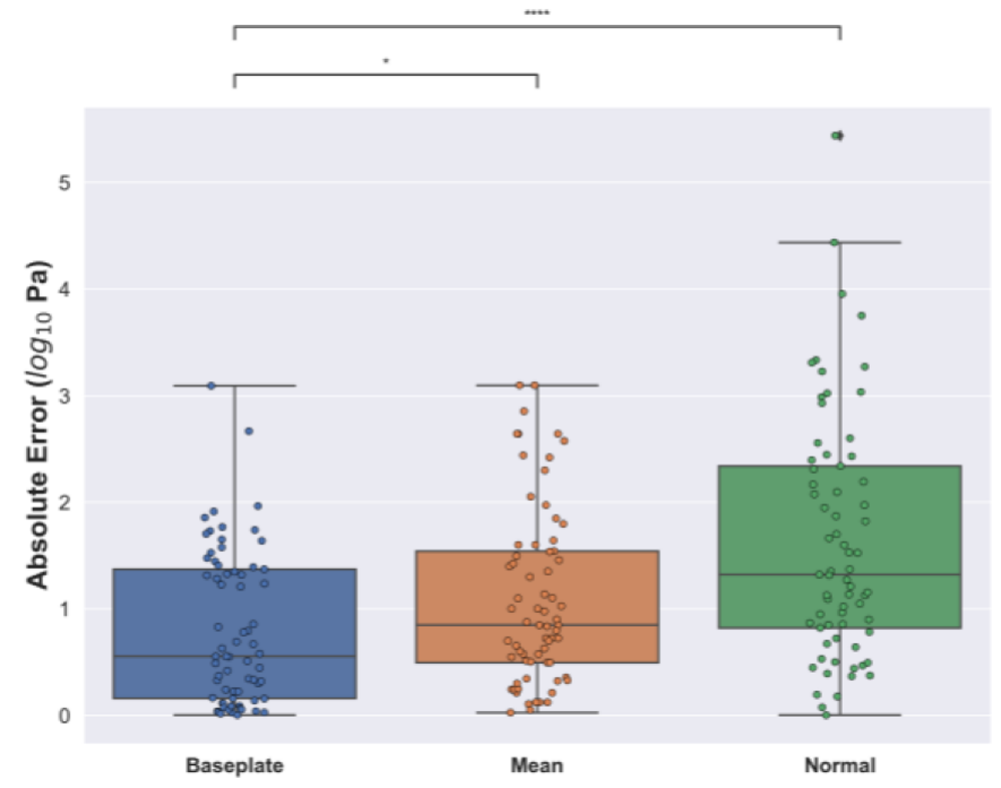
Box plot of absolute error produced by the baseplate, mean and normal model on the test set of the SEMPro data set, respectively (median, 25th, and 75th percentiles, minimum and maximum). [Legend: (*) *p* < 0.05, (****) *p* < 0.0001; paired, two-tailed *t*-test.]

We further conducted experiments with transfer learning, a machine learning approach that applies the knowledge acquired from solving one problem to another related problem, to further elucidate that our baseplate model can produce nontrivial results and demonstrate the transfer learning potential of the baseplate model for smaller hydrogel datasets. In the wholistic transfer learning experiment, we let the baseplate model predict the elastic moduli from a smaller dataset that it has not seen before (i.e., GO-PNIPAM dataset), and compared the corresponding MAE with the single and hybrid models, where the single and hybrid models are the ResNeXt50 and baseplate model trained on top of the GO-PNIPAM dataset, respectively. We also employed the Grad-CAM visualization technique to highlight the regions of the input SEM that are influential in the prediction. The original SEM images (resized and cropped to 224 × 224 pixels) were displayed in Figure 5a. The corresponding regression activation maps (Figures 5b–d), which utilize the gradients of the final convolutional layers in the model to emphasize localized areas of significance, show that our model produces nontrivial feature maps. Here, nontriviality is seen by the unique activation regions rather than repeated, nonstructure related motifs (i.e., the right/left edge of the image). The elastic modulus error (Δ*E*) of the prediction from each model is displayed under the respective regression activation map. Although the performance of the single model is relatively the same, compared to the hybrid model (Table S1 in the Supporting Information), the activation regions highlighted in Figure 5 are similar across hybrid, single, and baseplate models (see Figures 5b–d). Therefore, the wholistic transfer learning experiment indicates that our baseplate model extracts general features relevant to hydrogel SEMs, which can be transferred and targeted to specific hydrogels of interest. This is further demonstrated by our partial transfer learning approach (Table S2 in the Supporting Information), in which the baseplate model is still able to converge to a similar degree of performance as the hybrid and single models (Table S1) simply by training the output layer on the smaller, target dataset (i.e., GO-PNIPAM dataset). Therefore, the learned features of our baseplate model have a wholistic relevance to hydrogel SEMs that can be tuned to a given target hydrogel.

**Figure 5:**
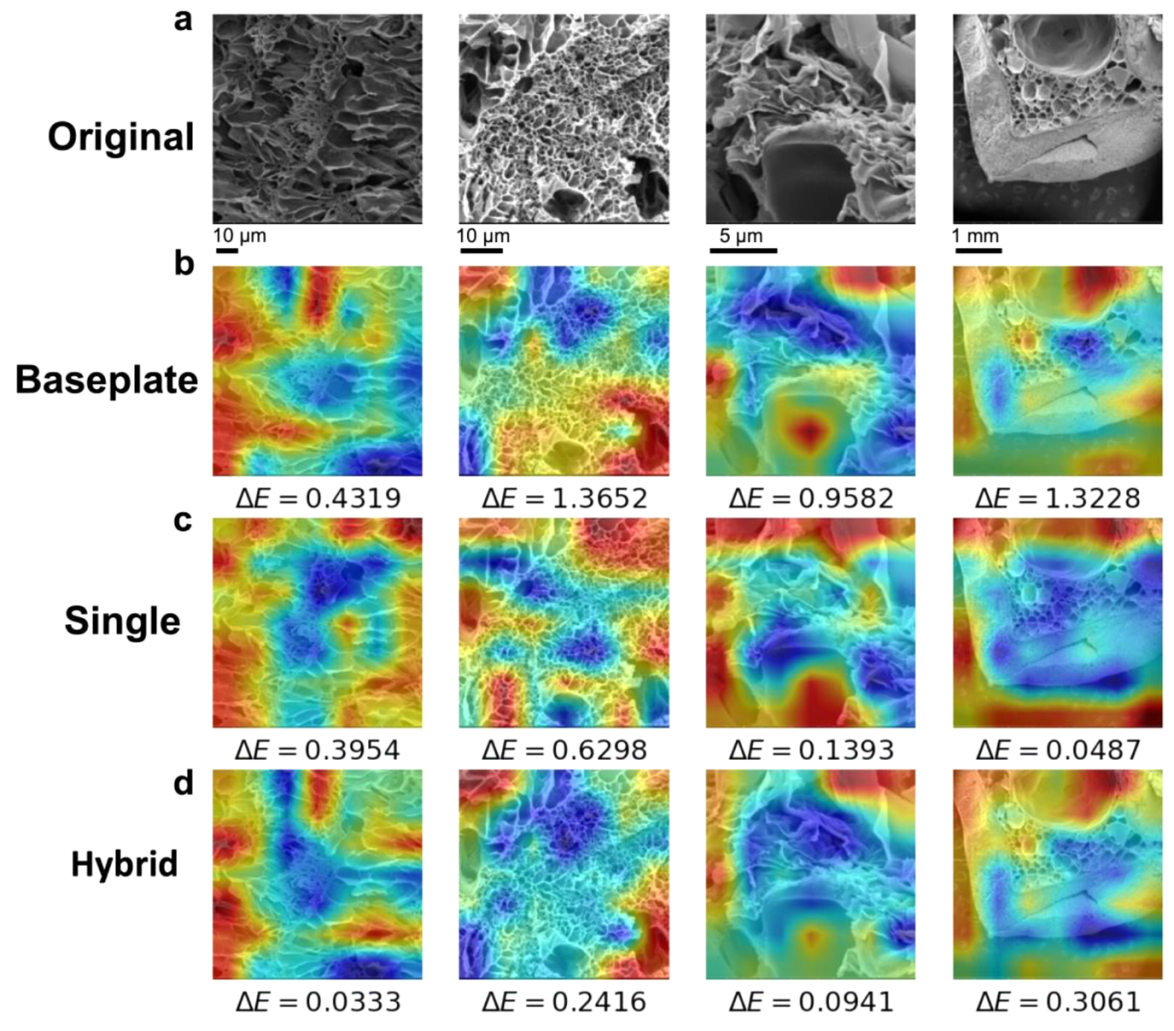
Examples of model performance and explainability on GO-PNIPAM hydrogel test dataset compared across variations in model training. (a) The original SEM images (resized and cropped to 224 × 224 pixels); The visual explanation maps for (b) baseplate, (c) single, and (d) hybrid model. The elastic modulus error (Δ*E*) of the prediction from each model is displayed under the respective regression activation map.

Our work introduced a new approach for performing efficient computational analysis on high-dimensional and sparse data in the field of materials science, specifically focusing on predicting elastic moduli from the SEM images of hydrogels. It is important to point out that the SEM images of hydrogels are typically taken after the hydrogel has been lyophilized and is in a dry state. Consequently, the reported porosities may not accurately reflect the hydrogel’s inherent porosity in the hydrated condition, as they can be influenced by ice crystal formation during lyophilization, introducing potential artifacts in SEM images during sample preparation. Although having artifact-free data for training deep learning models would be ideal, it is important to acknowledge the practical challenges in achieving this, as variations and errors are almost inevitable during the manufacturing process and necessary sample treatments for characterization. Our primary objective is to develop a tool capable of extracting valuable insights from typical SEM images, which often contain artifacts, and using this information to predict mechanical properties. To achieve this goal effectively, it is essential to work with an inclusive dataset that encompasses images with artifacts. This approach allows the model to learn and discern these artifacts as integral components of the “features” it extracts. One potential limitation of this study is that the model might encounter difficulties in accurately predicting results when presented with a nonporous SEM image of a hydrogel. This challenge primarily arises from the limited availability of similar data in the training dataset. To address this limitation, potential strategies include enlarging the training dataset by including more diverse data or implementing comprehensive validation and testing procedures. These procedures should involve evaluating the model’s performance on SEM images featuring nonporous structures, therefore suggesting a direction for future research.

In conclusion, this method offered a lightweight solution to analyze and extract valuable insights from the complex data structures commonly encountered in hydrogel research. In the data collection phase, we employed NLP techniques to scrape publicly available online journal repositories. This enabled us to gather a vast and diverse dataset of SEM images of hydrogels. The dataset was designed to be easily labeled by researchers within a short period, facilitating the availability of labeled data for subsequent analysis and model training. By leveraging NLP and automated data collection methods, we ensured the acquisition of a comprehensive and varied dataset for our research. With this dataset, we proved that we could train a model that is able to learn and extract features from our target material, hydrogels, relevant to our target metric, elastic moduli. Additionally, the model could be used for transfer learning on specific hydrogels and generating activation maps to explain the nontrivial predictions of the model. Through experimentation on a variety of hydrogels and model training methods, we showed that the learned features of our baseplate model were both valid and nontrivial. We also benchmarked the speed and performance of transfer learning achievable once a baseplate model was established. We hope that our pipeline would not only help researchers analyze hydrogel microstructure based on a larger, diverse dataset but also better utilize the repository of soft materials data in research papers for a greater understanding of high-dimensional, sparsely available metrics.

## Supporting Information Available

MAE for wholistic and partial transfer learning experiment (PDF)
