# Supplementary material for: SEMPro: A Data-Driven Pipeline To Learn Structure–Property Insights from Scanning Electron Microscopy Images
Source: ACS Mater Lett. 2023 Oct 24;5(11):3117–25. doi: 10.1021/acsmaterialslett.3c00909 (PMC10630981; doi:10.1021/acsmaterialslett.3c00909)
Supplement: Supplementary file 1 — tz3c00909_si_001.pdf [file tz3c00909_si_001.pdf]

Supporting Information

SEMPro: A Data-driven Pipeline to Learn Structure-Property Insights from Scanning Electron Microscopy Images

Brandon Ho[4‡]*, Jiayu Zhao*[1‡]*, Joseph Liu*[1]*, Lisa Tang*[1,2]*, Zhecun Guan*[1]*, Xiao Li*[3]*, Minghao Li*[3]*, Elizabeth Howard*[1]*, Rebecca Wheeler*[1,2]*, Jinhye Bae*[1,2,3*]

[1] Department of NanoEngineering, University of California San Diego, La Jolla, CA 92093, USA.

[2] Chemical Engineering Program, University of California San Diego, La Jolla, CA 92093, USA.

[3] Material Science and Engineering Program, University of California San Diego, La Jolla, CA 92093, USA.

[4] Department of Electrical and Computer Engineering, University of California San Diego, La Jolla, CA 92093, USA.

‡These authors contributed equally to this work.

[*]Corresponding author: j3bae@ucsd.edu

Table S1. MAE produced by baseplate, single, and hybrid in the wholistic transfer learning experiment.

	MAE		
	baseplate	single	hybrid
Fold 1	0.7908	0.3719	0.3246
Fold 2	0.8760	0.4027	0.3893
Fold 3	0.7061	0.3490	0.3579
Fold 4	0.9291	0.3953	0.4091
Fold 5	0.8810	0.3404	0.3298

Table S2. MAE produced by the baseplate and ResNeXt50 (from Pytorch pretrained model, trained on ImageNet) in the partial transfer learning experiment.

	MAE	
	Baseplate	ResNeXt50
Fold 1	0.4080	0.5639
Fold 2	0.4390	0.6839
Fold 3	0.4285	0.6952
Fold 4	0.5370	0.7717
Fold 5	0.4359	0.8653
